# Supplementary material for: HIVprotI: an integrated web based platform for prediction and design of HIV proteins inhibitors
Source: J Cheminform. 2018 Mar 9;10:12. doi: 10.1186/s13321-018-0266-y (PMC5845081; doi:10.1186/s13321-018-0266-y)
Supplement: Supplementary file 2 — Additional file 2. Source code of HIVProtI web server. [file 13321_2018_266_MOESM2_ESM.zip › HIVprotI_Source-code/hivprotI/weka-3-6-6/doc/weka/classifiers/trees/j48/C45PruneableClassifierTreeG.html]

C45PruneableClassifierTreeG


---


|  |  |  |
| --- | --- | --- |
| **Overview** \| **Package** \| **Class** \| **Tree** \| **Deprecated** \| **Index** \| **Help** \| **Weka's home** | |  |
| **PREV CLASS**   **NEXT CLASS** | **FRAMES**    **NO FRAMES**     **All Classes** |  |
| SUMMARY: NESTED \| FIELD \| CONSTR \| METHOD | DETAIL: FIELD \| CONSTR \| METHOD |  |


---


## weka.classifiers.trees.j48 Class C45PruneableClassifierTreeG

```
java.lang.Object
  weka.classifiers.trees.j48.ClassifierTree
      weka.classifiers.trees.j48.C45PruneableClassifierTreeG
```

**All Implemented Interfaces:**: java.io.Serializable, CapabilitiesHandler, Drawable, RevisionHandler

---

``` public class C45PruneableClassifierTreeG extends ClassifierTree ```

Class for handling a tree structure that can
be pruned using C4.5 procedures and have nodes grafted on.

**Version:**
:   $Revision: 5535 $

**Author:**
:   Janice Boughton (based on code by Eibe Frank)

**See Also:**: Serialized Form

---

**Field Summary**

**Fields inherited from interface weka.core.Drawable**

`BayesNet, Newick, NOT_DRAWABLE, TREE`


**Constructor Summary**

`C45PruneableClassifierTreeG(ModelSelection toSelectLocModel,
boolean pruneTree,
float cf,
boolean raiseTree,
boolean relabel,
boolean cleanup)`
  
          Constructor for pruneable tree structure.

`C45PruneableClassifierTreeG(ModelSelection toSelectLocModel,
Instances data,
ClassifierSplitModel gs,
boolean prune,
float cf,
boolean raise,
boolean isLeaf,
boolean relabel,
boolean cleanup)`
  
          Constructor for pruneable tree structure.


| **Method Summary** | |
| --- | --- |
| `double` | `biprob(double x, double n, double r)`             Significance test |
| `void` | `buildClassifier(Instances data)`             Method for building a pruneable classifier tree. |
| `void` | `collapse()`             Collapses a tree to a node if training error doesn't increase. |
| `void` | `doGrafting(Instances data)`             Initializes variables for grafting. |
| `Capabilities` | `getCapabilities()`             Returns default capabilities of the classifier tree. |
| `java.lang.String` | `getRevision()`             Returns the revision string. |
| `void` | `prune()`             Prunes a tree using C4.5's pruning procedure. |
| `void` | `setDescendents(java.util.ArrayList t, C45PruneableClassifierTreeG originalLeaf)`             add the grafted nodes at originalLeaf's position in tree. |
| `java.lang.String` | `toString()`             Prints tree structure. |

**Methods inherited from class weka.classifiers.trees.j48.ClassifierTree**

`assignIDs, buildTree, buildTree, classifyInstance, cleanup, distributionForInstance, graph, graphType, numLeaves, numNodes, prefix, toSource`

**Methods inherited from class java.lang.Object**

`equals, getClass, hashCode, notify, notifyAll, wait, wait, wait`

**Constructor Detail**

### C45PruneableClassifierTreeG

```
public C45PruneableClassifierTreeG(ModelSelection toSelectLocModel,
                                   boolean pruneTree,
                                   float cf,
                                   boolean raiseTree,
                                   boolean relabel,
                                   boolean cleanup)
                            throws java.lang.Exception
```

:   Constructor for pruneable tree structure. Stores reference
    to associated training data at each node.

    **Parameters:**: `toSelectLocModel` - selection method for local splitting model: `pruneTree` - true if the tree is to be pruned: `cf` - the confidence factor for pruning: `raiseTree` -: `cleanup` - **Throws:**: `java.lang.Exception` - if something goes wrong

---


### C45PruneableClassifierTreeG

```
public C45PruneableClassifierTreeG(ModelSelection toSelectLocModel,
                                   Instances data,
                                   ClassifierSplitModel gs,
                                   boolean prune,
                                   float cf,
                                   boolean raise,
                                   boolean isLeaf,
                                   boolean relabel,
                                   boolean cleanup)
```

:   Constructor for pruneable tree structure. Used to create new nodes
    in the tree during grafting.

    **Parameters:**: `toSelectLocModel` - selection method for local splitting model: `data` - the dta used to produce split model: `gs` - the split model: `prune` - true if the tree is to be pruned: `cf` - the confidence factor for pruning: `raise` -: `isLeaf` - if this node is a leaf or not: `relabel` - whether relabeling occured: `cleanup` - **Throws:**: `java.lang.Exception` - if something goes wrong


**Method Detail**

### getCapabilities

```
public Capabilities getCapabilities()
```

:   Returns default capabilities of the classifier tree.

    :   **Specified by:**: `getCapabilities` in interface `CapabilitiesHandler` **Overrides:**: `getCapabilities` in class `ClassifierTree`
    :   **Returns:**: the capabilities of this classifier tree **See Also:**: `Capabilities`

---


### buildClassifier

```
public void buildClassifier(Instances data)
                     throws java.lang.Exception
```

:   Method for building a pruneable classifier tree.

    :   **Overrides:**: `buildClassifier` in class `ClassifierTree`
    :   **Parameters:**: `datathe` - data for building the tree **Throws:**: `java.lang.Exception` - if something goes wrong

---


### collapse

```
public final void collapse()
```

:   Collapses a tree to a node if training error doesn't increase.

---


### prune

```
public void prune()
           throws java.lang.Exception
```

:   Prunes a tree using C4.5's pruning procedure.

    :   **Throws:**: `java.lang.Exception` - if something goes wrong

---


### doGrafting

```
public void doGrafting(Instances data)
                throws java.lang.Exception
```

:   Initializes variables for grafting.
    sets up limits array (for numeric attributes) and calls
    the recursive function traverseTree.

    :   **Parameters:**: `data` - the data for the tree **Throws:**: `java.lang.Exception` - if anything goes wrong

---


### setDescendents

```
public void setDescendents(java.util.ArrayList t,
                           C45PruneableClassifierTreeG originalLeaf)
```

:   add the grafted nodes at originalLeaf's position in tree.
    a recursive function that terminates when t is empty.

    :   **Parameters:**: `t` - the list of nodes to graft: `originalLeaf` - the leaf that the grafts are replacing

---


### biprob

```
public double biprob(double x,
                     double n,
                     double r)
              throws java.lang.Exception
```

:   Significance test

    :   **Parameters:**: `double` - x, double n, double r. **Returns:**: returns the probability of obtaining x or MORE out of n if r proportion of n are positive. z for normal estimation of binomial probability of obtaining x or more out of n, if r proportion of n are positive **Throws:**: `java.lang.Exception`

---


### toString

```
public java.lang.String toString()
```

:   Prints tree structure.

    :   **Overrides:**: `toString` in class `ClassifierTree`
    :   **Returns:**: the tree structure

---


### getRevision

```
public java.lang.String getRevision()
```

:   Returns the revision string.

    :   **Specified by:**: `getRevision` in interface `RevisionHandler` **Overrides:**: `getRevision` in class `ClassifierTree`
    :   **Returns:**: the revision


---


|  |  |  |
| --- | --- | --- |
| **Overview** \| **Package** \| **Class** \| **Tree** \| **Deprecated** \| **Index** \| **Help** \| **Weka's home** | |  |
| **PREV CLASS**   **NEXT CLASS** | **FRAMES**    **NO FRAMES**     **All Classes** |  |
| SUMMARY: NESTED \| FIELD \| CONSTR \| METHOD | DETAIL: FIELD \| CONSTR \| METHOD |  |


---
